# Comment on "Comparison of the effect of cardioplegic solutions used in cardiac surgery on myocardial protection"

**DOI:** 10.1590/1806-9282.20250741

**Published:** 2025-10-27

**Authors:** Hüseyin Durmaz

**Affiliations:** 1 Konya City Hospital – Konya, Turkey.

Dear Editor,

I read with interest the article titled "Comparison of the effect of cardioplegic solutions used in cardiac surgery on myocardial protection,"^1^ recently published in the journal Revista da Associação Médica Brasileira by Dr. Padak et al. The study is significant in terms of emphasizing myocardial protection.

The data presented in the study clearly demonstrate the characteristics of the solutions used for myocardial protection and the differences between them. In this sense, the suggestions presented to increase myocardial protection performance are quite important. However, I think there are some points that need to be addressed in this study.

It is an undeniable fact that there is no ideal cardioplegia technique for myocardial protection. I think it is important for the study to be homogeneous, as the patient group, evaluated as patients undergoing cardiac surgery, did not state which cardiac surgery they underwent. This should be taken into consideration, as the extent of cardiac enzyme changes may vary significantly between patients with coronary artery disease and those without.

The method of using cardioplegic solution in myocardial protection may change the success of myocardial protection, but it is seen that the study does not state whether cardioplegia is given antegrade or retrograde.

There are many factors that can cause an increase in the acute-phase reactant known as C-reactive protein. Considering that cardiac surgery patients are usually comorbid patients, I think that C-reactive protein can be affected by various factors.

In conclusion, the study by Dr. Padak et al. offers a valuable perspective by addressing an important issue in cardiac surgery. I eagerly await further research in this area and hope that the authors will consider these suggestions in their future studies.

Thank you for the opportunity to provide feedback on this important study.

Yours truly

## Data Availability

The datasets generated and/or analyzed during the current study are available from the corresponding author upon reasonable request.
